# Editorial: The Impact of Active and Passive Smoking upon Health and Neurocognitive Function

**DOI:** 10.3389/fpsyt.2016.00148

**Published:** 2016-08-29

**Authors:** Tom Heffernan

**Affiliations:** ^1^Department of Psychology, Northumbria University, Newcastle upon Tyne, UK

**Keywords:** active smoking, passive smoke exposure, health, neurocognitive function, smoking cessation

**The Editorial on the Research Topic**

**The Impact of Active and Passive Smoking upon Health and Neurocognitive Function**

Tobacco smoking is a major risk factor for a number of chronic diseases, including a variety of cancers, lung disease, and damage to the cardiovascular system. The World Health Organization recently calculated that there were six million smoking-attributable deaths per year and that this number is due to rise to about eight million per year by the end of 2030. Recent work has demonstrated that habitual smoking in adults is associated with a range of health conditions, including cardiovascular disease, pulmonary dysfunction, and an increased risk of a variety of cancers. In terms of neurocognitive function, although some studies have found that acute smoking can enhance cognitive functions in the short term, actually chronic smoking is deleterious in the long term. Chronic smoking has been associated with reductions in working memory (the temporary storage and manipulation of information), executive function (planning tasks, focusing ones attention, and ignoring irrelevant distractions), and prospective memory (memory for everyday things, such as keeping an appointment, or taking an important medication on time). More recently, the focus on smoking-related health problems and neurocognitive deficits has expanded to include the study of “second-hand smoking” (also known as “passive smoking” – wherein a person who does not smoke him/herself inhales tobacco smoke either via side-stream smoke or via smoke being blown directly into his/her face). Research in this area has linked exposure to second-hand smoke in those who have never smoked to a range of health problems akin to smokers, including lung and cardiovascular disease, as well as deficits in neurocognitive function. In terms of neurocognitive function, exposure to second-hand smoke has been linked with an increased risk of mild cognitive impairments in older adults, reductions in working memory, as well as deficits in executive function. Interventions aimed at reducing cigarette consumption and improving the health of both smokers and those exposed to second-hand smoke continue to be developed. The aim of this Frontiers Research Topic is to bring together a collection of papers that look at what impact active and passive smoking has upon health and neurocognitive function; as well as to consider some of the wide variety of interventions aimed at reducing cigarette use and/or improving health.

Copeland examined pre-treatment characteristics among daily smokers (including smoking patterns, smoking outcome expectancies, and smoking-related health information) and how these related to success on a brief motivational enhancement intervention. Marshall et al. explored whether the combined (polydrug) effect of consuming excessive amounts of alcohol and smoking cigarettes exacerbated everyday memory problems when compared with the sum of their independent effects (excessive drinking alone, or smoking alone). Philibert et al. examined whether aryl hydrocarbon receptor repressor (AHRR) can be used to determine whether AHRR methylation status is a quantifiable biomarker for progress in smoking cessation that could have substantial impact on both smoking cessation treatment and research. Ling and Heffernan reviewed evidence in relation to the cognitive consequences of exposure to second-hand smoke in those who had no history of smoking. Payne et al. evaluated chronic obstructive pulmonary disease-related health factors in flight attendants exposed to second-hand cigarette smoke and assessed whether meditative movement was effective as a treatment in improving pulmonary function in these flight attendants. Jukosky et al. demonstrated how cigarette exposure alters the innate immune response and increases an individual’s susceptibility to pathogen infection when compared with non-exposed individuals. Jovanovic and Jakovljevic discuss regulatory control of e-cigarette composition and raises concern regarding the quality control and health outcomes surrounding e-cigarettes. The commentary by Parrott discusses concerns about the paradoxical nature of using e-cigarettes; whether they may in fact be damaging to physical/psychological health of the users, as well as raising concerns about what impact e-cigarettes have upon those who are “passively vaping.” Lasebikan and Ola assessed the efficacy of screening, brief intervention, and referral for treatment package to reduce tobacco smoking in two semi-rural community settings in South-West Nigeria.

Overall, the papers presented in this *Frontiers in Psychiatry* special topic demonstrates the broad nature of research currently being undertaken in relation to active and passive smoking and some of the current issues surrounding the use of e-cigarettes as nicotine-replacement therapy. The research cited here should pave the way for further work in this area. Areas for future research include the concern of what impact exposure to second-hand smoke might be having upon children’s health, neurocognitive function, and educational achievement, an area of particular importance given the recent estimates from the World Health Organization that approximately 40% of children across the world are regularly exposed to second-hand smoke in the home. A further area that has received very little attention at all is whether exposure to “third-hand smoke” (the residue of nicotine and other chemicals left on indoor surfaces as a result of tobacco) smoking has a detrimental impact upon those who have never smoked, both in terms of health and neurocognitive function.

## Author Contributions

This is the sole work of the author and editor for this Special Topic.

## Conflict of Interest Statement

The author declares that the research was conducted in the absence of any commercial or financial relationships that could be construed as a potential conflict of interest.

